# Navigating Digital Dermatology: An Analysis of Acne-Related Content on TikTok

**DOI:** 10.7759/cureus.45226

**Published:** 2023-09-14

**Authors:** Bilal Irfan, Ihsaan Yasin, Aneela Yaqoob

**Affiliations:** 1 Microbiology & Immunology, University of Michigan, Ann Arbor, USA; 2 Electrical Engineering and Computer Science, University of Michigan, Ann Arbor, USA; 3 Infectious Diseases, Beaumont Hospital, Taylor, USA

**Keywords:** content quality, acne, social media education, social media analytics, tiktok

## Abstract

Background

With TikTok’s rising popularity as a hub for health information dissemination, the quality and nature of such content require assessment. This study investigates the popularity and quality of the top 100 most-liked videos tagged with “#acne” on TikTok. This study aims to examine the engagement and quality of acne-related content on TikTok, assess contributions from diverse sources, including physicians and non-physicians, and guide healthcare professionals in leveraging this platform for public health education.

Methodology

A cross-sectional analysis of the top 100 most-liked videos tagged with “#acne” on TikTok as of June 7, 2023, was conducted. Parameters assessed included the profession of the creator, gender, specialty, content type, and other observable characteristics. The quality was measured using the DISCERN tool.

Results

Of the dataset, 38 videos were by physicians and 29 by non-physicians. Physician-created content had higher mean views, likes, comments, shares, and favorites than non-physician-created content. Videos by dermatologists and non-dermatologists received similar engagement. Videos sharing personal experiences achieved the highest DISCERN score. Overall, DISCERN scores were uniformly low across all categories.

Conclusions

Physicians, especially dermatologists, are trusted sources of acne-related information on TikTok. The study underscores the need for professionals to provide reliable, evidence-based information on such platforms, guiding effective health communication in the digital age.

## Introduction

TikTok, a rapidly growing social media platform, has become more than just a destination for entertainment and creative expression; it has evolved into a space where individuals across the globe seek information on various subjects, including health-related topics like acne [1,2]. With over two billion downloads worldwide, TikTok offers an interactive, engaging, and immediate connection to diverse audiences, especially among younger generations [3]. Its widespread reach and visual appeal have created new channels for healthcare information dissemination and engagement, contributing to an unprecedented shift in how people access, consume, and share health information.

Acne, a common dermatological condition affecting millions worldwide, has significant social and emotional ramifications [4-7]. Often perceived as a cosmetic issue, acne can lead to decreased self-esteem, anxiety, and even depression, making public education and access to accurate information critically important [8]. The surge in interest in acne-related content on social media platforms like TikTok highlights the contemporary need to understand, manage, and treat this pervasive condition.

The popularity and ubiquity of social media platforms have radically altered the landscape of health information access [9,10]. Where traditional sources such as medical journals, clinics, and healthcare providers were once the primary channels of information, platforms like TikTok now enable immediate access to a wide array of perspectives [11]. This democratization of information offers both opportunities and challenges. While healthcare professionals can reach a broader audience, there is also a risk of misinformation, especially when content is produced by non-experts [12].

TikTok’s user-friendly interface and the ability to generate viral content have made it a preferred platform for many seeking quick, visual, and engaging information [13]. Its algorithm, promoting user interaction, may lead to a preference for easily digestible content over scientifically rigorous information, thus affecting the quality of health-related material found on the platform [14].

This study aims to examine the popularity and quality of the top 100 most-liked videos tagged with “#acne” on TikTok, comparing contributions from various sources, including physicians and non-physicians, and assessing the influence of factors such as physician specialty, gender, and the nature of experience shared in the videos. In a world where misinformation can spread quickly, assessing the quality and impact of such widely consumed content is crucial. Understanding the landscape of acne-related content on TikTok can guide healthcare professionals, educators, and policymakers in leveraging this platform effectively to foster public health education, particularly concerning acne awareness, prevention, and treatment.

## Materials and methods

The study’s objective was to assess the popularity and quality of the top 100 most-liked videos tagged with “#acne” on TikTok as of June 7, 2023. To achieve this, a cross-sectional analysis was conducted, focusing on various parameters to provide a comprehensive view of the content. The selection criteria were predefined to ensure relevance and consistency. Videos were included if they were tagged with “#acne” and were among the top 100 most-liked videos on the platform as of the specified date. Exclusion criteria included videos that were not primarily in English, were shorter than 10 seconds, or had been removed from the platform.

The parameters assessed for each video included the profession of the creator (physician, non-physician, private company, etc.), gender, specialty (if applicable), content type (e.g., personal experiences, home remedies, educational content, treatment advertising), and other observable characteristics. Data were manually extracted and recorded in a standardized spreadsheet. The information included video views, likes, comments, shares, favorites, and specific metadata related to video content and creator.

The quality of health information was assessed using the DISCERN tool, a validated 15-point questionnaire that evaluates health information concerning treatment options, risks, benefits, and other aspects [15]. Two independent reviewers assigned scores from 1 (low) to 5 (high) for each video using the DISCERN tool. Discrepancies between reviewers were resolved through averaging their scores.

## Results

The dataset included 38 videos by physicians and 29 videos by non-physicians. Physician-created content had higher mean views, likes, comments, shares, and favorites than non-physician-created content. The findings in Table 1 indicate that videos by physicians are generally more popular and this trend may reflect the public’s demand for credible health information online.

**Table 1:** Overview of acne content popularity and quality on TikTok.

Content demographics	Number of videos (%)	Mean number of views	Mean number of likes	Mean number of comments	Mean number of shares	Mean number of favorites	Mean DISCERN scores
Profession							
Physicians	38	712,370	16,395	243	566	1,779	1.25505
Non-physicians	29	312,317	9315	83	821	1,543	1.22472
Private companies	27	614,091	24,196	322	1,300	2,444	1.23856
Other health professionals	6	846,417	13,045	23	55	87	1.2775
Gender							
Male	53	523,245	9,948	129	305	1,182	1.23982
Female	45	590,108	15,396	181	825	1,773	1.247978
Other designation	2	614,091	24,196	322	1,300	2,444	1.2386
Physician specialty							
Dermatologists specialist	23	706,113	16,350	246	292	1,455	1.25396
Non-dermatologists specialist	15	721,962	16,463	239	988	2,275	1.25673
Experience							
Personal experience	16	668,250	14,504	164	189	775	1.261813
Home remedies	14	570,121	14,513	107	1,305	3,272	1.23743
Educational content	17	435,204	13,567	232	302	1,506	1.23453
Treatment advertising	53	598,378	18,090	235	1,025	1,793	1.2418

The observed data offers an intriguing insight into the preferences and behaviors of TikTok users seeking acne-related content. Physicians’ videos have almost double the views and likes compared to non-physicians, underlining a preference for content created by medical professionals. Surprisingly, private companies hold a position closer to physicians in terms of views and likes, which may be indicative of a certain level of trust in commercial solutions or a reflection of brand influence. Other health professionals, though presumably experts in the field, trail behind all others, pinpointing a clear preference for physicians in the perception of viewers.

In examining the gender distribution of the content creators, a more nuanced picture emerges. Female creators tend to have higher mean views, likes, and favorites, although the differences are not overly pronounced. This finding could be indicative of societal interests or possibly point to content style and approach differences based on gender. However, the relatively small divergence in these statistics necessitates caution in attributing significant gender influence on content popularity.

## Discussion

Similar results in engagement across physician specialties suggest that the audience may not strongly differentiate between dermatologists and non-dermatologist physicians when seeking acne information, with their DISCERN scores aligning closely as well. This implies a level of trust and reliance on the credibility of the medical profession as a whole, rather than a specific specialty.

When considering the nature of content and its resonation with the audience, several key insights arise. Videos sharing personal experiences, though numerically fewer, achieve the highest DISCERN score. This may underscore that sharing personal experiences could reflect the perceived authenticity and relatability of such content. It emphasizes the importance of narrative medicine and the incorporation of personal stories in health education efforts [16-22]. Conversely, the popularity of home remedies and treatment advertising content, despite only medium-level DISCERN scores, highlights an area that requires caution. It suggests a public interest in alternative treatments or commercial solutions that may not always align with scientific evidence [23,24]. Educational content’s lower views and likes might suggest that there remains an unmet need to make such content more engaging or accessible.

The gender dynamics in the content popularity present a more complex picture. Although female creators tend to achieve higher engagement, the differences are subtle. It prompts further investigation into content style, approach, and societal interests that may influence these trends. Additionally, private companies’ close alignment with physicians in engagement metrics raises questions about commercial influence or possible trust in branded content.

Perhaps the most striking observation from this analysis is the uniformly low DISCERN scores across all categories and demographics. These scores, ranging narrowly from 1.22472 to 1.2775, emphasize a systemic issue in the quality of acne-related information provided on TikTok. This pattern points to a significant area of concern and opportunity. Health professionals, particularly physicians, could leverage this platform to deliver more evidence-based, comprehensive, and reliable information [25].

Limitations

A comprehensive understanding of the study’s findings necessitates acknowledging several potential limitations that may have influenced the results. The ever-changing and dynamic nature of TikTok’s content may affect the consistency and repeatability of the findings. As videos on the platform are continually uploaded, updated, and removed, the representativeness of the dataset at any given moment may be limited. This dynamic nature affects the generalizability of the study’s conclusions and may introduce variability in the observed trends.

The methodology used in the selection of videos might also introduce selection bias, limiting the study’s scope. Specific keywords, algorithms, and search criteria may have skewed the representation of content categories, gender distribution, professional affiliations, or other variables under study. For example, only #acne was searched, and other videos that may relate to the topic without the given hashtag were not explored. Searches conducted with #acnevulgaris or other combinations of words such as #acnetreatment may have postulated different results. Furthermore, the validity of the DISCERN tool employed to assess video content quality has been questioned for this specific context [26]. Differences in video format, presentation style, and multimedia elements might not be adequately captured by the tool, hindering a nuanced evaluation of content quality. Analyzing the use of such platforms is relevant as telehealth is being explored as a means to deliver culturally conscious care [27].

Other considerations include the influence of the time of posting a given video and the geographic region from which the search was conducted. These temporal and spatial variables might introduce additional variability in video visibility and engagement metrics. Such factors could confound the analysis, leading to an incomplete understanding of content popularity and effectiveness.

The study’s focus on TikTok may also limit its applicability to other social media platforms or content channels. The unique characteristics and user demographics of TikTok might influence content engagement and popularity in ways that are not generalizable to other online mediums. Moreover, while the study provides insights into apparent trends and patterns, it does not delve into the underlying factors influencing these trends or their subsequent impacts on viewer behaviors. The absence of deeper analysis into motivational, psychological, sociocultural, or other contextual factors limits the understanding of why certain content types, creator categories, or thematic elements resonate differently with viewers.

## Conclusions

The popularity of #acne content on TikTok underscores a broader societal trend in which individuals increasingly seek health information on social media platforms. This study sheds light on the preferences and behaviors of viewers in relation to acne-related content and highlights the critical role of various creators, such as physicians, private companies, and others in shaping public understanding. Physicians, particularly those specializing in dermatology, emerge as trusted voices in the realm of acne-related content, reflecting both public trust in medical professionals and the resonance of scientifically grounded information. This observation signals an opportunity for medical professionals to leverage this platform strategically, disseminating reliable, evidence-based, acne-related information. By harnessing TikTok’s vast reach and engaging style, physicians can contribute to enhancing public health education and awareness, aligning content with scientific evidence, and fostering an informed public discourse.

The study’s findings, considered within the context of its potential limitations, offer a starting point for understanding the complex interplay between content creators, viewers, platform dynamics, and health information quality. It invites further exploration into the unique characteristics of social media as a vehicle for health communication, the evolution of public health messaging in the digital age, and the imperative for targeted interventions that align with emerging trends. Collectively, these insights contribute to an evolving field of inquiry that has implications for health communication strategies, public health policy, medical education, and clinical practice, marking a significant step in aligning the digital information landscape with evidence-based healthcare objectives.
